# Preconception Mental Health, Socioeconomic Status, and Pregnancy Outcomes in Primiparous Women

**DOI:** 10.3389/fpubh.2022.880339

**Published:** 2022-07-13

**Authors:** Satu-Maarit Björkstedt, Hannu Koponen, Hannu Kautiainen, Mika Gissler, Pirjo Pennanen, Johan G. Eriksson, Merja K. Laine

**Affiliations:** ^1^Department of General Practice and Primary Health Care, University of Helsinki and Helsinki University Hospital, Helsinki, Finland; ^2^Social Services and Health Care Division, Helsinki, Finland; ^3^Department of Psychiatry, University of Helsinki and Helsinki University Hospital, Helsinki, Finland; ^4^Folkhälsan Research Center, Helsinki, Finland; ^5^Primary Health Care Unit, Kuopio University Hospital, Kuopio, Finland; ^6^Finnish Institute for Health and Welfare, Helsinki, Finland; ^7^Karolinska Institute and Region Stockholm, Stockholm, Sweden; ^8^Vantaa Health Center, Vantaa, Finland; ^9^Department of Obstetrics and Gynecology and Human Potential Translational Research Program, Yong Loo Lin School of Medicine, National University Singapore, Singapore, Singapore; ^10^Agency for Science, Technology and Research (A^*^STAR), Singapore Institute for Clinical Sciences (SICS), Singapore, Singapore

**Keywords:** mental illness, preconception psychiatric diagnosis, pregnancy outcomes, primiparous, socioeconomic status

## Abstract

**Background:**

One in four women of childbearing age has some degree of mental disorders and are, therefore, prone to both pregnancy complications and adverse health outcomes in their offspring. We aimed to evaluate the impact of preconception severe mental disorders on pregnancy outcomes in primiparous women.

**Methods:**

The study cohort was composed of 6,189 Finnish primiparous women without previously diagnosed diabetes, who delivered between 2009 and 2015, living in the city of Vantaa, Finland. Women were classified to have a preconception severe mental disorder if they had one or more outpatient visits to a psychiatrist or hospitalization with a psychiatric diagnosis 1 year before conception. Data on pregnancies, diagnoses, and pregnancy outcomes were obtained from national registers at an individual level.

**Results:**

Primiparous women with preconception severe psychiatric diagnosis were younger, more often living alone, smokers, and had lower educational attainment and lower taxable income than women without psychiatric diagnosis (for all *p* < 0.001). Of all women, 3.4% had at least one psychiatric diagnosis. The most common psychiatric diagnoses were depression and anxiety disorders. The most common comorbidity was the combination of depression and anxiety disorders. There were no differences in the need for respiratory treatments, admissions to the neonatal intensive care unit, or antibiotic treatments between the offspring's groups.

**Conclusion:**

Although primiparous women had severe mental disorders, the well-being of newborns was good. The most common severe mental health disorders were depression and anxiety disorders, and psychiatric comorbidity was common. Women with severe mental disorders more often belonged to lower socioeconomic groups.

## Introduction

Around one in one-quarter of women of childbearing age have mental disorders (1–4). The prevalence of mental disorders in young pregnant women is increasing (5). Of the preconception mental disorders, depression and anxiety are most common (4, 6). Comorbidity is common for mental disorders (7, 8). The poor preconception of mental health is a risk factor for both pregnancy complications and adverse health outcomes in the offspring (9–14).

A population-based US study reported that poor preconception mental health was the most significant risk factor for pregnancy complications and was reported in every third pregnancy. Women who reported poor mental health before pregnancy were 1.48 times more likely to have a non-live birth, and nearly twice as likely to give birth to a low-birthweight baby. Overall, pregnancy complications were identified in four out of ten women (10). Studies, including both primiparous and multiparous women, show that women with preconception common mental disorders (CDM, i.e., depression and anxiety) are more likely to have pregnancy complications, such as gestational diabetes mellitus (GDM), miscarriage or stillbirth, and delivery of a preterm or a low-birthweight newborn (9–11). A recent systematic review and meta-analysis show that antenatal maternal anxiety was associated with preterm birth and low birthweight, but not with pre-eclampsia, and cesarean delivery (11). Most of these studies were from North America and Europe and the adverse outcomes of offspring were associated with socioeconomic characteristics, such as young or old maternal age, smoking, and low education (9–11). However, the study results are conflicting regarding preconception anxiety and depression and their association with GDM (1, 13, 15, 16). Different definitions (interviews, purchases of drugs, registered data, and self-reported) of anxiety, depression, and GDM, as well as different study designs (cross-sectional, longitudinal) and study populations, make comparisons between studies challenging.

In pregnant women, mental disorders are associated with socioeconomic status (SES) (12, 14, 17, 18). In most studies, SES is associated with maternal age, smoking, educational attainment, income, and cohabiting (12, 14). Smoking is more frequent among women, who are unmarried and/or have low educational attainment (14, 18).

In primiparous women, data on preconception mental health and its influence on pregnancy complications and outcomes are largely missing. The purpose of this study is to evaluate maternal mental health and morbidity and their association with pregnancy outcomes in primiparous women.

## Materials and Methods

The study cohort included all primiparous women (*N* = 6189) without previously diagnosed diabetes mellitus from the city of Vantaa, Finland, who delivered a singleton between January 1, 2009 and December 31, 2015, and who spoke Finnish or Swedish as their native language. Vantaa is the fourth most populated city in Finland located in Helsinki metropolitan area. We excluded women with diabetes mellitus to avoid the independent association with common mental disorders (CMD) (1, 15, 16).

Women were defined to be primiparous if they delivered for the first time a live birth or a stillbirth from 500 g or 22 gestational weeks onward.

The Finnish Institute of Health and Welfare (THL) keeps the Finnish Medical Birth Register, including data on all births and stillbirths in Finland. From this source, we obtained the following maternal characteristics: age at delivery, the status of cohabiting, smoking, pre-pregnancy weight and height, pregnancy history (number of previous pregnancies, abortions, miscarriages, and ectopic pregnancies), presence of GDM, hospitalization due to hypertension or vaginal bleeding, cesarean section, and duration of pregnancy. The day of conception was a subtraction of the date of delivery and the duration of pregnancy.

Women were defined as smokers if they smoked or quit smoking during pregnancy. Pre-pregnancy body mass index (BMI) was calculated as a quotient of pre-pregnancy weight (kg) and height (m) squared. The threshold value of BMI for obesity was ≥30 kg/m^2^. Diagnosis of chronic diseases was obtained from The Finnish Institute of Health and Welfare (THL).

Since 2008, the diagnoses of GDM are assessed according to the Finnish Current Care Guidelines for GDM (www.kaypahoito.fi) with the following threshold glucose values in a standard 75 g 2-h oral tolerance test (OGTT): fasting plasma glucose ≥ 5.3 mmol/l, 1-h glucose ≥10 mmol/l, 2-h glucose ≥8.6 mmol/l. One or more pathological values lead to the diagnosis of GDM.

The level of education was defined as the highest level of education achieved. This data was obtained from Statistics Finland. Education was distributed into four levels: I basic education (9–10 years of schooling), II upper secondary education/ postsecondary non-tertiary education (11–14 years of schooling), III bachelor's degree education (15–16 years of schooling), and IV master's/doctoral education (≥17 years of schooling).

The Finnish Tax Administration produces yearly data on taxable income, including both earned and capital income at the individual level. The pre-pregnancy annual taxable incomes of primiparous women were defined as the mean taxable income for the year of consumption and 2 years before delivery. The annual incomes were adjusted for the year 2017 value by a consumer price index (Statistics Finland http://www.stat.fi/til/index¬_en.html). The study population was divided into five annual income levels (euros, €) I level 0–11 120 €, level II 11 121–22 855 €, level III 22 856–29 940 €, level IV 29 941–40 190 €, and level V 40 191 € or more. The mean annual taxable income in Finland among women in 2016 was 24 764 €.

The Finnish Institute of Health and Welfare (THL) also keeps the Care Register for Health Care. This register contains data on information of patients in inpatient care and health centers, patients discharged from inpatient care, day surgeries, and specialized outpatient care. The diagnosis was coded according to the International Statistical Classification of Diagnosis and Related Health Problems 10th Revision (ICD-10). World Health Organization 1992. From the Care Register for Health Care, we obtained data on maternal psychiatric diagnosis (ICD-10 code group F, excluding F00–F10 because there were no cases within these groups) 1 year before conception. Women were classified as having a mental health disorder if they had one or more outpatient psychiatrist visit/s or hospitalization due to mental health disorders. The onset of mental disorders was defined as the day the psychiatric diagnosis was coded.

Based from the Finnish Medical Birth Register, we also obtained the following offspring characteristics: sex, birth weight and height, head circumference and admissions to neonatal intensive care treatment, resuscitation with intubation, antibiotic treatment, and respiratory treatment before the age of 7 days. Offspring birthweight was calculated as Z-scores according to sex and gestational age within our own cohort. The ponderal index was calculated by dividing the birth weight (kg) by the birth length (m)^3^ (19).

Preterm birth was defined as a delivery before 37+0 gestational weeks. Cesarean sections were divided into emergency, urgent, and elective sections.

### Statistical Analyses

The characteristics are presented as means with standard deviation (SD) for continuous variables and as frequencies with percentages for categorical variables. Statistical comparison between the study participants with and without a psychiatric diagnosis was performed by the *t*-test, Chi-square test, or Fisher–Freeman–Halton test when appropriate. Prevalence of preconception psychiatric diagnoses among primiparous women according to ICD-10 codes per 1,000 person-years with 95% confidence intervals (CI) was calculated assuming a Poisson distribution. Offspring birthweight was calculated as Z-scores according to sex and gestational age. In the case of violation of the assumptions (e.g., non-normality) for continuous variables, a bootstrap-type method or Monte Carlo p-values (small number of observations) for categorical variables were used. No adjustment was made for multiple testing because this information can be obtained by multiplying the actual *p*-value by the number of comparisons made (20). There was no statistical difference in the main outcome variables, so multiple testing would not have changed the results. Normal distributions were evaluated graphically and with the Shapiro–Wilk W test. Stata 17.0 (StataCorp LP, College Station, TX, USA) was used for the analysis.

## Results

The mean age of the study participants was 28.6 (SD 5.1) years. Women with a psychiatric diagnosis (ICD-10 code group F) were younger (25.3 [5.7] years vs. (28.6 [5.1] years), more often living alone (40% vs. 20%), less often non-smokers (70% vs. 90%), had lower educational attainment (11.4 years vs. 13.5 years), and lower yearly income (12 700 vs. 25 000) than women without a psychiatric diagnosis, for all *p* < 0.001. Table 1 shows the characteristics of women without and with psychiatric diagnoses. Women without psychiatric diagnoses needed more often hospitalization due to hypertension during pregnancy compared with women with a psychiatric diagnosis (7% vs. 2%, *p* = 0.013). Table 2 shows the pregnancy complications in women without and with a psychiatric diagnosis.

**Table 1:** Characteristics of primiparous women (*N* = 6189) without and with preconception psychiatric diagnosis.

	**Primiparous women**		**P–value**
	**Without psychiatric diagnosis (n = 5979)**	**With psychiatric diagnosis (n = 210)**	
Age (years), mean (SD)	28.6 (5.1)	25.3 (5.7)	<0.001
Cohabiting, *n* (%)	4797 (80)	127 (60)	<0.001
Yearly income 1000, mean (SD)	25.0 (13.7)	12.7 (10.5)	<0.001
Smokers, n (%)			<0.001
No smoking	4927 (82)	120 (57)	
Quit during the 1st trimester	462 (8)	26 (12)	
Smoking during pregnancy	590 (10)	64 (30)	
Years of schooling, mean (SD)	13.5 (2.6)	11.4 (2.4)	<0.001
Height (cm), mean (SD)	166 (6)	166 (6)	0.49
Weight (kg), mean (SD)	66 (14)	68 (14)	0.085
Pre–pregnancy body mass index (kg/m^2^), mean (SD)	24.0 (4.5)	24.8 (5.0)	0.028
Obese (body mass index ≥30.0 kg/m^2^), *n* (%)	648 (11)	32 (15)	0.045
Morbidity, *n* (%)			
Thyroid diseases	40 (1)	0 (0)	0.65
Rheumatic diseases	74 (1)	2 (1)	0.99
Pulmonary diseases	182 (3)	9 (4)	0.31
Inflammatory bowel diseases	54 (1)	3 (1)	0.44
Cardiovascular diseases	4 (0)	0 (0)	0.99
Epilepsy	43 (1)	4 (2)	0.074

**Table 2:** Pregnancy complications in primiparous women (*N* = 6189) without and with preconception psychiatric diagnosis.

	**Primiparous women**		**P–value**
	**Without psychiatric diagnosis (n = 5979)**	**With psychiatric diagnosis (n = 210)**	
Previous pregnancies, *n* (%)			0.036
None	4801 (80)	159 (76)	
One	837 (14)	29 (14)	
Two	256 (4)	16 (8)	
Three or more	85 (1)	6 (3)	
Fertility treatment, *n* (%)	537 (9)	6 (3)	0.002
Duration of pregnancy (weeks), mean (SD)	39.9 (1.9)	39.7 (2.3)	0.099
Gestational diabetes mellitus, *n* (%)	962 (16)	41 (20)	0.18
Hospitalization due to hypertension, *n* (%)	389 (7)	5 (2)	0.013
Hospitalization due to vaginal bleeding, *n* (%)	21 (0)	0 (0)	0.99
Cesarean sections, *n* (%)			0.34
Emergency	86 (7)	6 (11)	
Urgent	908 (70)	38 (70)	
Elective	310 (24)	10 (19)	
Preterm delivery, *n* (%)	350 (6)	16 (8)	0.29
Epidural pain relief, *n* (%)	4032 (67)	145 (69)	0.62

### The Psychiatric Diagnosis

**Figure 1:**
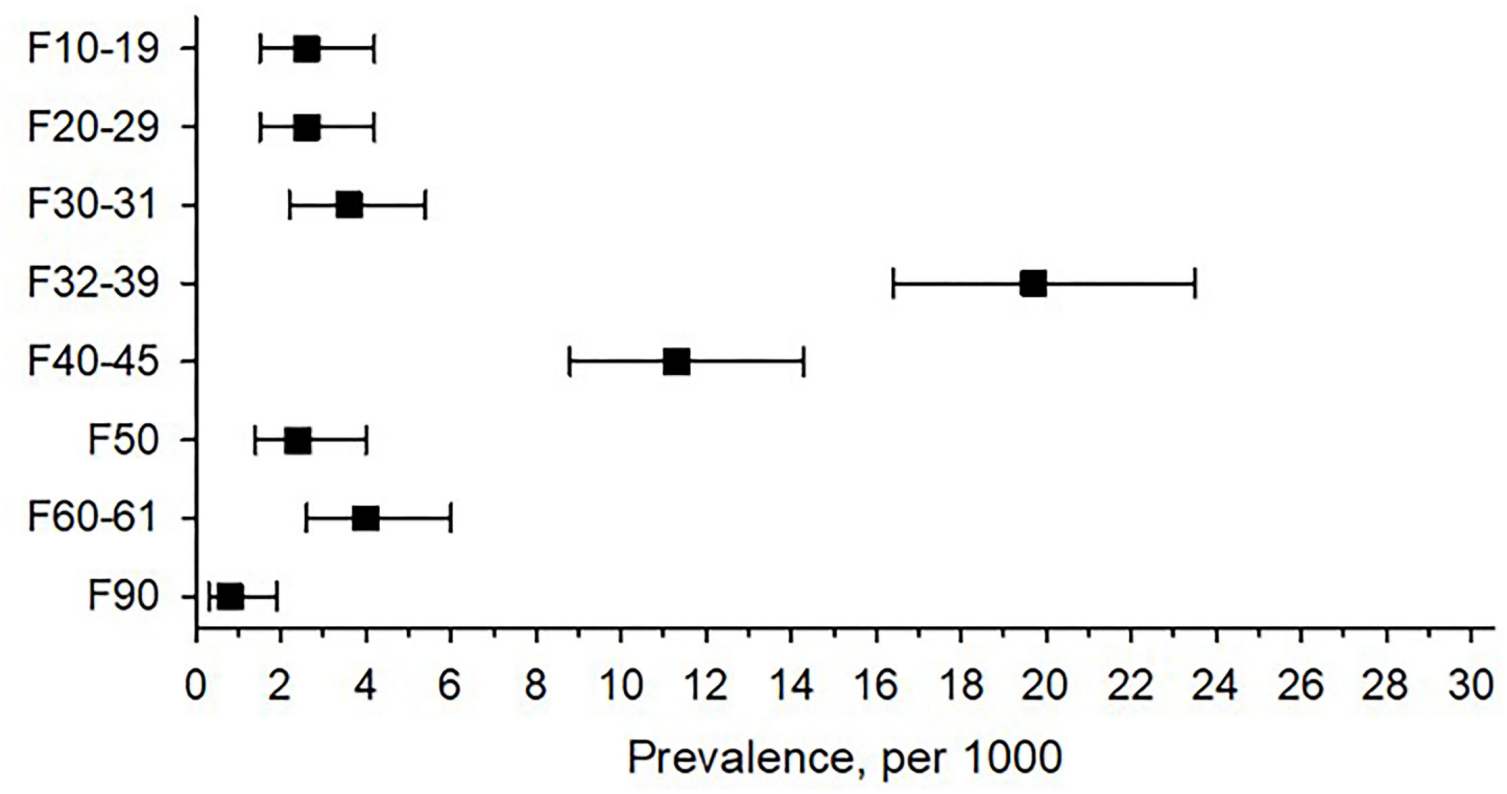
Prevalence of preconception psychiatric diagnoses among primiparous women according to International Statistical Classification of Diagnosis and Related Health Problems 10th Revision (ICD-10) codes. ICD 10 = International Statistical Classification of Diseases and Related Health Problems F10–19 mental and behavioral disorders due to psychoactive substance use, F20–29 schizophrenia, schizotypal and delusional disorders, F30–31 bipolar affective disorders, F32–39 depressive disorders, F40–45 anxiety disorders, F50 eating disorders, F60–61 disorders of adult personality and behavior, and F90 disturbance of activity and attention.

One year before conception, 3.4% (*n* = 210) of women had at least one psychiatric diagnosis. The most common psychiatric diagnosis was depression (ICD-10 codes F32–39), observed in 20 out of 1,000 primiparous women (95% CI 16.4,23.4). The second most common psychiatric diagnosis was anxiety disorder (ICD-10 codes F40–45), observed in 11 out of 1,000 women (95% CI 8.8,14.3). Figure 1 shows the prevalence of psychiatric diagnosis according to the ICD-10 codes in primiparous women.

### Psychiatric Comorbidity

One year before conception in primiparous women, 10 out of 1,000 (95% CI 7.8,13.0) had more than one psychiatric diagnosis. The most common psychiatric comorbidity was depression (ICD-10 codes F32-−39) and anxiety disorders combined (ICD-10 codes F40–45), this was observed in 4 out of 1,000 women (95% CI 2.7,6.1). The second most common psychiatric comorbidity was anxiety disorder (ICD-10 codes F40–45) and alcohol and/or narcotics abuse (ICD-10 codes F10–19) combined, which was observed in 1 out of 1,000 women (95% CI .4,2.1). The psychiatric comorbidity with depression (ICD-10 codes F32–39) and abuse of alcohol and/or narcotics (ICD-10 codes F10–19) were observed in 1 out of 1,000 women (95% CI 0.3, 1.9).

### Psychiatric Diagnosis and Pregnancy Outcomes

Table 3 shows pregnancy outcomes in primiparous women divided into women without and with psychiatric diagnoses diagnosed 1 year before conception. Among offspring in these groups, no differences were observed in birth weight or birth length or in the need for respiratory treatments, admissions to neonatal intensive care unit, or antibiotic treatments.

**Table 3:** Pregnancy outcomes in primiparous women (*N* = 6189) without and with preconception psychiatric diagnosis.

	**Primiparous women**			**P–value**
	**Without psychiatric diagnosis (n = 5979)**	**With psychiatric diagnosis (n = 210)**	**Crude**	Adjusted^*^
Male, *n* (%)	3113 (52)	97 (46)	0.094	0.061
Birth weight (g), mean (SD)				
Male	3471 (556)	3502 (665)	0.58	0.52
Female	3369 (547)	3320 (557)	0.35	0.45
Z–score	−0.00 (1.00)	0.06 (0.98)	0.34	0.17
Length (cm), mean (SD)				
Male	50.2 (2.5)	50.0 (3.7)	0.30	0.58
Female	49.4 (2.7)	49.2 (3.0)	0.26	0.49
Ponderal index (kg/m^3^)	27.4 (2.6)	27.8 (2.8)	0.051	0.31
Head circumference (cm), mean (SD)	34.8 (1.8)	34.6 (2.1)	0.12	0.44
Respiratory treatment	96 (2)	6 (3)	0.16	0.32
Admission to neonatal intensive care unit	656 (11)	28 (13)	0.28	0.68
Antibiotic treatment	394 (7)	19 (9)	0.16	0.64

* *Adjusted with maternal age, cohabiting, yearly income, smoking, years of schooling, and pre–pregnancy body mass index (BMI)*.

## Discussion

Primiparous women with severe preconception psychiatric diagnosis more often belonged to lower socioeconomic groups and shared several characteristics of poor SES. Of women in the study population, 3% had at least one psychiatric diagnosis. The most common psychiatric diagnoses were depression and anxiety disorders. Further, 1% of the women had more than one preconception psychiatric diagnosis. The most common psychiatric comorbidity was depression and anxiety disorders combined. Between offspring of women with preconception psychiatric diagnosis and offspring of women without a psychiatric diagnosis were no differences in the need for respiratory treatments, admissions to neonatal intensive care unit, or antibiotic treatments.

We found that primiparous women with severe mental disorders had low SES. Previous studies have shown that smoking is more frequent among women, who are unmarried and/or have low educational attainment (14, 18). One study including nulliparous women focused on their health literacy. Women with inadequate health literacy were younger, had less educational attainment, and publicly funded insurance compared with those with adequate health literacy (20). Married women had a lower risk for low-birthweight neonates (10). In the US, the strongest protective issue against pregnancy complications was public or private health insurance (10). On the other hand, SES had little impact on adverse birth outcomes, although low maternal education weakly increased the likelihood of a low-birthweight neonate (12). There are conflicting results over the impact of maternal SES in pregnancy and outcomes. In many studies, there is heterogeneity of cohorts and the associations between variations remain unclear (11, 21, 22). In the study, the prevalence of GDM in this study was 16% as expected. ln 2015, the overall prevalence of GDM in Finland was 16% (18).

On average, one in five adults experiences common mental disorders within 1 year, and 30% over the lifespan. Women had higher rates of mood (7% vs. 4%) and anxiety (9% vs. 4%) disorders than men, while men had higher rates of substance use disorders (7% vs. 2%) than women (23). Further, women from low-, middle-, or high-income countries had more mood and anxiety disorders than men (24). In Finland, the yearly prevalence of depression is 5–7% (8). According to a survey by World Mental Health (WMH), the prevalence of anxiety disorders is 10% (25). We found that 3% of women had at least one psychiatric diagnosis. Our low prevalence rates are explained, at least in part, by the fact that the available diagnosis was made by psychiatrists in an outpatient clinic or hospital department. In Finland, the patients who visit the outpatient clinic or department have difficult-to-treat symptoms and/or a more severe illness. Patients with mild diseases are generally treated in primary health care centers by general practitioners.

We observed that the comorbidity of depression, anxiety disorders, and substance abuse disorders was common. According to a systematic review focusing on the prevalence of comorbid mental illness in clients with substance use, it varied from 47% to 100% (7). In Finland, the prevalence of comorbidity of depression and substance abuse varies between 10% and 30% (8). Further, the prevalence of comorbidity of anxiety disorders and substance abuse for 1 year is 5–30%, and during the lifespan 20–40% (8).

Generally, hypertensive disorders complicate 5–0% of all pregnancies (26). We found that women without a psychiatric diagnosis were hospitalized due to hypertension more often than women with a h psychiatric diagnosis. Possibly, women with a psychiatric diagnosis are closely monitored, and potential blood pressure problems are detected at an early stage when hospitalization is not required. Further, women with a psychiatric diagnosis were younger.

We observed no differences in newborns' weight, length, or admissions to neonatal intensive care unit, and respiratory or antibiotic treatments during the first week of life between offspring of women without and with preconception psychiatric diagnosis. In Finland, antenatal care in maternity clinics is comprehensive. Of all pregnant women, 99.7% use maternity clinic services (https://thl.fi/fi/web/lapset-nuoret-ja-perheet/sote-palvelut/aitiys-ja-lastenneuvola/aitiysneuvola). The maternity clinic gives counseling pregnant women individually and special attention is paid to high-risk mothers. Another study reported no difference between women taking psychotropic drugs compared with a group taking no medication in gestational age and birth weight (27). We were unaware of the prenatal medication for the women, which may explain the differences in the study observations. In our study cohort, women with mental disorders had low SES. Previous studies have shown controversial findings between low SES measured by income and educational attainment and the risk of preterm birth, low birth weight, and need for neonatal intensive care unit treatments (11, 12, 21, 22).

We recognize several strengths in our study. Our study cohort included all primiparous women who fulfilled the inclusion criteria, from the 4th biggest city in Finland with 220,000 inhabitants in 2015. We utilized the Finnish medical registers which are comprehensive, valid, and high-quality (28). A personal identification number assigned for every citizen and permanent resident since the 1960s enables us to combine data from different national registers. Further, the psychiatric diagnosis was made by trained psychiatrists.

There are some limitations to our study. Although our study cohort was comprehensive, all our study participants were Finnish. Thus, the generalization of the results is limited. Although our study cohort was large, there were few serious psychiatric diagnoses, which should be kept in mind when evaluating our research findings. We evaluated maternal preconception mental health and pregnancy outcomes, but we are unaware the medication used during pregnancy.

In conclusion, newborn of primiparous women with severe mental diseases seem to be born in good health. Primiparous women with severe mental health disorders often come from lower SES groups. Preconception prevalence of severe mental diseases was low, and comorbidity was common.

## Data Availability Statement

The datasets generated for this study are not publicly available due to legal limitations.

## Ethics Statement

The studies involving human participants were reviewed and approved by the Ethics Committee of the Hospital District of Helsinki and Uusimaa, Finland (356/13/03/03/2015), November 2, 2015), and the health authority of the city of Vantaa, Finland. The following registers have given permission to use register data in the study: Finnish Institute for Health and Welfare (THL), The Finnish Social Insurance Institution and Statistics Finland. The patients/participants provided their written informed consent to participate in this study.

## Author Contributions

S-MB wrote the manuscript. S-MB, HKa, MG, JE, and ML contributed to the study design, data collection, and research data. S-MB, HKo, HKa, MG, PP, JE, and ML contributed to the interpretation of the results and to the discussion, reviewed the article critically, and approved the final version of the manuscript. All authors contributed to the article and approved the submitted version.

## Funding

Research Foundation for Primary Health Care, Finland, and The Hospital District of Helsinki and Uusimaa, Finland, awarded a research grant for this study. The funding sources had no role in the preparations of this article nor in study design, data collection, analyses, or writing the article.

## Conflict of Interest

The authors declare that the research was conducted in the absence of any commercial or financial relationships that could be construed as a potential conflict of interest.

## Publisher's Note

All claims expressed in this article are solely those of the authors and do not necessarily represent those of their affiliated organizations, or those of the publisher, the editors and the reviewers. Any product that may be evaluated in this article, or claim that may be made by its manufacturer, is not guaranteed or endorsed by the publisher.

## References

[1] Beka Q Bowker L Savu A Kingston D Johnson JA Kaul P. History of mood and anxiety disorders and risk of gestational diabetes mellitus in a population-based cohort. Diabet Med. (2018) 3:147–151. 10.1111/dme.13543 29120506

[2] United Nations World Population Prospects 2019 File FERT/4: Total fertility by region subregion and country, 1950-2100 (live births per woman) Estimates 1950 – 2020 POP/DB/WPP/Rev.2019/FERT/F04

[3] Global regional and national incidence prevalence and years lived with disability for 354 diseases and injuries for 195 countries and territories 1990–2017: 1990–2017: a systematic analysis for the Global Burden of Disease Study 2017 GBD 2017 disease and injury incidence and prevalence collaborators*. Lancet. (2018) 392:1789–858. 10.1016/S0140-6736(18)32279-7 30496104 PMC6227754

[4] Faramarzi M Kheirkhah F Barat S Cuijpers P O'Connor E Ghadimi R et al. Prevalence and factors related to psychiatric symptoms in low-risk pregnancy. Caspian J Intern Med. (2020) 11:211–8. 10.22088/cjim.11.2.211 32509251 PMC7265507

[5] Howard LM Khalifeh H. Perinatal mental health: a review of progress and challenges. World Psychiatry. (2020) 19:313–27. 10.1002/wps.20769 32931106 PMC7491613

[6] Ban L Gibson JE West J Fiaschi L Oates MR Tata LJ. Impact of socioeconomic deprivation on maternal perinatal mental illnesses presenting to UK general practice. Br J Gen Pract. (2012) 62:e671–8. 10.3399/bjgp12X656801 23265226 PMC3459774

[7] Kingston REF Marel C Mills KL. A systematic review of the prevalence of comorbid mental health disorders in people presenting for substance use treatment in Australia. Drug Alcohol Rev. (2017) 36:527–39. 10.1111/dar.12448 27786426

[8] Working group set by the Finnish Medical Society Duodecim the Medical Advisory Board of the Finnish Psychiatric Association. Depression and anxiety: Current Care Guidelines. Available online at: www.kaypahoito.fi (accessed depression 11 March 2021).

[9] Spry EA Wilson CA Middleton M Moreno-Betancur M Doyle LW Howard LM et al. Parental mental health before and during pregnancy and offspring birth outcomes: A 20-year preconception cohort of maternal and paternal exposure. EClinicalMedicine. (2020) 27:100564. 10.1016/j.eclinm.2020.100564 33150327 PMC7599306

[10] Witt WP Wisk LE Cheng ER Hampton JM Hagen EW. Preconception mental health predicts pregnancy complications and adverse birth outcomes: a national population-based study. Matern Child Health J. (2012) 16:1525–41. 10.1007/s10995-011-0916-4 22124801 PMC3605892

[11] Grigoriadis S Graves L Peer M Mamisashvili L Tomlinson G Vigod SN et al. Maternal anxiety during pregnancy and the association with adverse perinatal outcomes: systematic review and meta-analysis. J Clin Psychiatry. (2018) 79:17r12011. 10.4088/JCP.17r12011 30192449

[12] Campbell EE Gilliland J Dworatzek PDN De Vrijer B Penava D Seabrook JA. Socioeconomic status and adverse birth outcomes: A population-based canadian sample. J Biosoc Sci. (2018) 50:102–13. 10.1017/S0021932017000062 28270256

[13] Baugh N Harris DE Aboueissa AM Sarton C Lichter E. The impact of maternal obesity and excessive gestational weight gain on maternal and infant outcomes in maine: analysis of pregnancy risk assessment monitoring system results from 2000 to 2010. J Pregnancy. (2016) 2016:5871313. 10.1155/2016/587131 27747104 PMC5055984

[14] Ospina M Osornio-Vargas ÁR Nielsen CC Crawford S Kumar M Aziz K et al. Socioeconomic gradients of adverse birth outcomes and related maternal factors in rural and urban Alberta, Canada: a concentration index approach. BMJ Open. (2020) 10:e033296. 10.1136/bmjopen-2019-033296 32014876 PMC7045252

[15] Wilson CA Santorelli G Dickerson J Ismail K Reynolds RM Simonoff E et al. Is there an association between anxiety and depression prior to and during pregnancy and gestational diabetes? an analysis of the born in bradford cohort. J Affect Disord. (2020) 276:345–50. 10.1016/j.jad.2020.07.019 32741755 PMC7477491

[16] Wilson CA Newham J Rankin J Ismail K Simonoff E Reynolds RM et al. Is there an increased risk of perinatal mental disorder in women with gestational diabetes? Meta-Analysis Diabet Med. (2020) 37:602–22. 10.1111/dme.14170 31693201 PMC7154542

[17] Cruz-Bendezú AM Lovell GV Roche B Perkins M Blake-Lamb TL Taveras EM et al. Psychosocial status and prenatal care of unintended pregnancies among low-income women. BMC Pregnancy Childbirth. (2020) 20:615. 10.1186/s12884-020-03302-2 33046003 PMC7552564

[18] Rönö K Masalin S Kautiainen H Gissler M Raina M Eriksson JG et al. Impact of maternal income on the risk of gestational diabetes mellitus in primiparous women. Diabet Med. (2019) 36:214–20. 10.1111/dme.13834 30307050

[19] Landmann E Reiss I Misselwitz B Gortner L. Ponderal index for discrimination between symmetric and asymmetric growth restriction: percentiles for neonates from 30 weeks to 43 weeks of gestation. J Matern Fetal Neonatal Med. (2006) 19:157–60. 10.1080/14767050600624786 16690508

[20] Hochberg MC. A sharper Bonferroni procedure for multiple test of significance. Biometria. (1988) 75:800–2. 10.1093/biomet/75.4.800

[21] Yee LM Silver R Haas DM Parry S Mercer BM Wing DA et al. Association of health literacy among nulliparous individuals and maternal and neonatal outcomes. JAMA Netw Open. (2021) 4:e2122576. 10.1001/jamanetworkopen.2021.22576 34468757 PMC8411280

[22] Dunlop AL Essalmi AG Alvalos L Breton C Camargo CA Cowell WJ et al. program collaborators for environmental influences on child health outcomes. racial and geographic variation in effects of maternal education and neighborhood-level measures of socioeconomic status on gestational age at birth: Findings from the ECHO cohorts. PLoS ONE. (2021) 16:e0245064. 10.1371/journal.pone.0245064 33418560 PMC7794036

[23] Steel Z Marnane C Iranpour C Chey T Jackson JW Patel V et al. The global prevalence of common mental disorders: a systematic review and meta- analysis 1980-2013. Int J Epidemiol. (2014) 43:476–93. 10.1093/ije/dyu038 24648481 PMC3997379

[24] Fisher J Cabral de Mello M Patel V Rahman A Tran T Holton S et al. Prevalence and determinants of common perinatal mental disorders in women in low- and lower-middle-income countries: a systematic review. Bull World Health Organ. (2012) 90:139G−49. 10.2471/BLT.11.091850 22423165 PMC3302553

[25] Alonso J Liu Z Evans-Lacko S Sadikova E Sampson N Chatterji S et al. WHO world mental health survey collaborators. treatment gap for anxiety disorders is global: results of the World Mental Health Surveys in 21 countries. Depress Anxiety. (2018) 35:195–208. 10.1002/da.22711 29356216 PMC6008788

[26] Luger RK Kight BP. Hypertension in Pregnancy-StatPearls. Treasure Island (FL): StatPearls Publishing (2021). 28613589

[27] Michielsen LA van der Heijden FM Janssen PK Kuijpers HJ. Effects of maternal psychotropic drug dosage on birth outcomes. Neuropsychiatr Dis Treat. (2014) 10:13–8. 10.2147/NDT.S53430 24376355 PMC3865140

[28] Gissler M. Finnish health and social welfare registers in epidemiological research. Norsk Epidemiologi. 14:113–20. 10.1002/da.2271

